# The factors influencing inappropriate child feeding practices among families receiving nutrition allowance in the Himalayan region of Nepal

**DOI:** 10.1186/s40795-023-00691-3

**Published:** 2023-02-20

**Authors:** Dev Ram Sunuwar, Anuradha Bhatta, Anjana Rai, Narendra Kumar Chaudhary, Man Kumar Tamang, Suvash Nayaju, Devendra Raj Singh

**Affiliations:** 1 Department of Nutrition and Dietetics, Nepal Armed Police Force Hospital, Kathmandu, Nepal; 2 Department of Public Health, Asian College for Advance Studies, Purbanchal University, Lalitpur, Nepal; 3 School of Public Health and Social Work, Queensland University of Technology, Brisbane City, Australia; 4 Department of Radiology, Nepal Orthopaedic Hospital, Jorpati, Kathmandu, Nepal; 5 Queensland Brain Institute, The University of Queensland, Brisbane, Australia; 6 School of Human and Health Sciences, University of Huddersfield, Huddersfield, UK; 7 Research Section, Swadesh Development Foundation, Siraha, Nepal

**Keywords:** Child cash grant, Child feeding practices, Cash transfers, Nutrition allowance, Nepal

## Abstract

**Background:**

Child feeding practices during the first two years of life are crucial to ensure good health and nutrition status. This study aimed to assess the factors influencing inappropriate child feeding practices in children aged 6 − 23 months in families receiving nutrition allowance in the remote Mugu district, Nepal.

**Methods:**

A community-based cross-sectional study was conducted among 318 mothers who had children aged 6 − 23 months of age in the seven randomly selected wards. Systematic random sampling technique was used to select the desired number of respondents. Data were collected using pre-tested semi-structured questionnaire. Bivariate and multivariable binary logistic regression was used to estimate crude odds ratio (cOR), and adjusted odds ratio (aOR), and 95% confidence intervals (CIs) to understand factor associated with child feeding practices.

**Results:**

Almost half of the children aged 6 − 23 months were not consuming a diverse diet (47.2%; 95% CI: 41.7%, 52.7%), did not meet the recommended minimum meal frequency (46.9%; 95% CI: 41.4%, 52.4%) and did not consume minimum acceptable diet (51.7%; 95% CI: 46.1%, 57.1%). Only 27.4% (95% CI: 22.7%, 32.5%) of children met the recommended complementary feeding practices. Multivariable analysis showed maternal characteristics such as mothers who gave birth at home (aOR = 4.70; 95% CI: 1.03, 21.31) and mothers in unpaid employment (aOR = 2.56; 95% CI: 1.06, 6.19) were associated with increased odds of inappropriate child feeding practices. Household economy (i.e. family with < 150 USD monthly income) was also associated with increased odds of inappropriate child feeding practices (aOR = 1.19; 95% CI: 1.05, 2.42).

**Conclusion:**

Despite the receipt of nutritional allowances, child feeding practices among 6 − 23 months children were not optimal. Additional context-specific behavior change strategies on child nutrition targeting mothers may be required.

**Supplementary Information:**

The online version contains supplementary material available at 10.1186/s40795-023-00691-3.

## Introduction

Adequate nutrition during the first year of life and early childhood is crucial to ensure sustainable health outcomes for children [1]. Despite the unanimous global and national efforts made through a plethora of nutrition policies and interventions [2], child malnutrition remains a major global public health challenge [3]. Nearly half (45%) of childhood deaths are estimated to be associated with poor nutrition status [4]. As of 2021, it was estimated that 49 million children were wasting and over 149 million children under the age of five were stunted [4]. Child undernutrition cuts down future economic productivity by at least 20% [5]. Subsequently, malnutrition could decrease Gross Domestic Product (GDP) by 2 − 11% [6, 7]. The evidence suggests that investments in children now will contribute toward a healthier and more educated workforce that is better able to contribute to the country’s economy [6, 7].

The first two years of life are a critical window of opportunity for the prevention of undernutrition through optimal child feeding practices [6, 8]. For the last one and a half decade, improving child feeding practices, particularly for children under two years of age, has been a global priority [6]. However, the Infant and Young Child Feeding (IYCF) practices are not satisfactory and do not meet the optimum recommended guidelines [9]. Particularly in resource-poor countries, several challenges contribute to non-compliance with IYCF practices [1]. In Africa and South Asia, almost half of all the children in the poorest households were not meeting the recommended minimum meal frequency and the minimum diet diversity [9] due to wealth-related inequalities and poor translation of policy into practices [10, 11].

In Nepal, improving children's nutritional status is a national priority which has led to considerable improvements in recent years [12]. However, undernutrition causes 25,000 child deaths in Nepal each year, accounting for 52% of all child deaths higher than any other cause of childhood death [13]. Between 2001 and 2022, Nepal reduced stunting by 56.2% to 25% [14]. Despite these remarkable improvements, undernutrition remains unacceptably high in Nepal, with a stunting prevalence reported at 25%, and wasting at 8% in 2022, with a prevalence of double and triple malnutrition of 6.6% and 7.0%, respectively [14, 15]. About 36% of them are from poor households with a total per capita consumption of less than US$ 180 [12]. A large proportion of Nepalese households also have food insecurity and poor dietary diversity [16, 17]. The causes of childhood malnutrition are complex and are associated with several demographic, economic, clinical, social, and environmental factors [3]. The UNICEF framework of childhood undernutrition identifies the i) individual-level immediate causes such as infection, dietary intake; ii) household-level underlying causes such as household food insecurity, child-caring practices; and iii) environmental-level basic causes such as access to education, health services, and economic and socio-cultural context [18].

### Nutrition allowance scheme in Nepal

The Government of Nepal implemented the nutrition allowance scheme (locally known as *Poshan Bhataa*) programme as a strategy to prevent malnutrition and improve nutrition status among children. Nepal’s nutrition allowance programme provides cash transfers to households with children under five years of age in eight districts of Karnali province (*Humla, Jumla, Mugu, Dolpa, Kalikot, Bhajang,* and *Aacham*) and one district in province 2 (*Rautahat*) [19]. The nutrition allowance scheme programme started in the fiscal year 2009/2010 and continued till date [20]. Under the programme policy, the local government provided (Nepalese rupees) NRs 200 (~ 2 USD) per month to the mother or primary caregiver of eligible children, and later in 2018, the allowance was doubled to NRs 400 (~ 4 USD) [21]. The allowance was provided for up to two children under five years of age in a household [20].

Nepal’s cash transfer programme, however, is programmatically different from the conditional cash transfer (CCT) programme which has emerged as an effective intervention to address both the underlying and the immediate causes of childhood malnutrition [22, 23]. The purpose of both types of transfers is to provide social protection to support poor households in accessing health and nutrition services. Nepal’s cash transfer programme is expected to have a measurable impact on reducing child malnutrition rates through improving better child feeding practices by providing households with additional resources to meet food, health, and care needs [24].

A previous study has examined the impact assessment of Nepal’s unconditional nutrition allowance on childhood anthropometry [23], although current evidence on how unconditional cash transfer influences child feeding practices in Nepal is sparse. This study aimed to explore current child feeding practices and assess factors influencing feeding practices of children aged 6 − 23 months in families receiving nutrition allowance in the Mugu district of Nepal.

## Methods

### Study design and setting

A community-based cross-sectional study was conducted in *Chhayanath Rara* urban municipality in the Mugu district of Nepal from April 2021 to December 2021. The data were collected between May 5 and July 1, 2021. Mugu district lies in Karnali Province in the Himalayan region at an elevation of 1,201 to 6,717 m and borders China to the north. The district has very limited roadways and infrastructure and health care facilities are poor due to geographical remoteness [25]. The *Chhayanath Rara* urban municipality has a population of 20,078 [26].

The participants of this study were mothers with children 6 − 23 months of age. Mothers who were receiving a nutrition allowance and consented to participate in this study were included. Mothers with cognitive impairment, disability, and non-consent were not eligible to participate in this study.

### Sample size determination and sampling strategy

The sample size was calculated using the single population proportion formula for finite population:

$$\mathrm{n}=\frac{\left[{z}^{2}\mathrm{p }\left(1-\mathrm{p}\right)/{\mathrm{e}}^{2}\right]}{1+\left[{z}^{2}\mathrm{p }\left(1 -\mathrm{p}\right)/{\mathrm{e}}^{2}\mathrm{N}\right]}$$

A total of 1,154 mother–child pairs were used as a finite population (N) [27]. The proportion (p) was considered 50%, with margin error (e) at 5%, and standard normal deviation (Z) at a confidence limit of 95%. The total sample size was 318 with the addition of a 10% non-response rate to the calculated sample size.

**Fig. 1:**
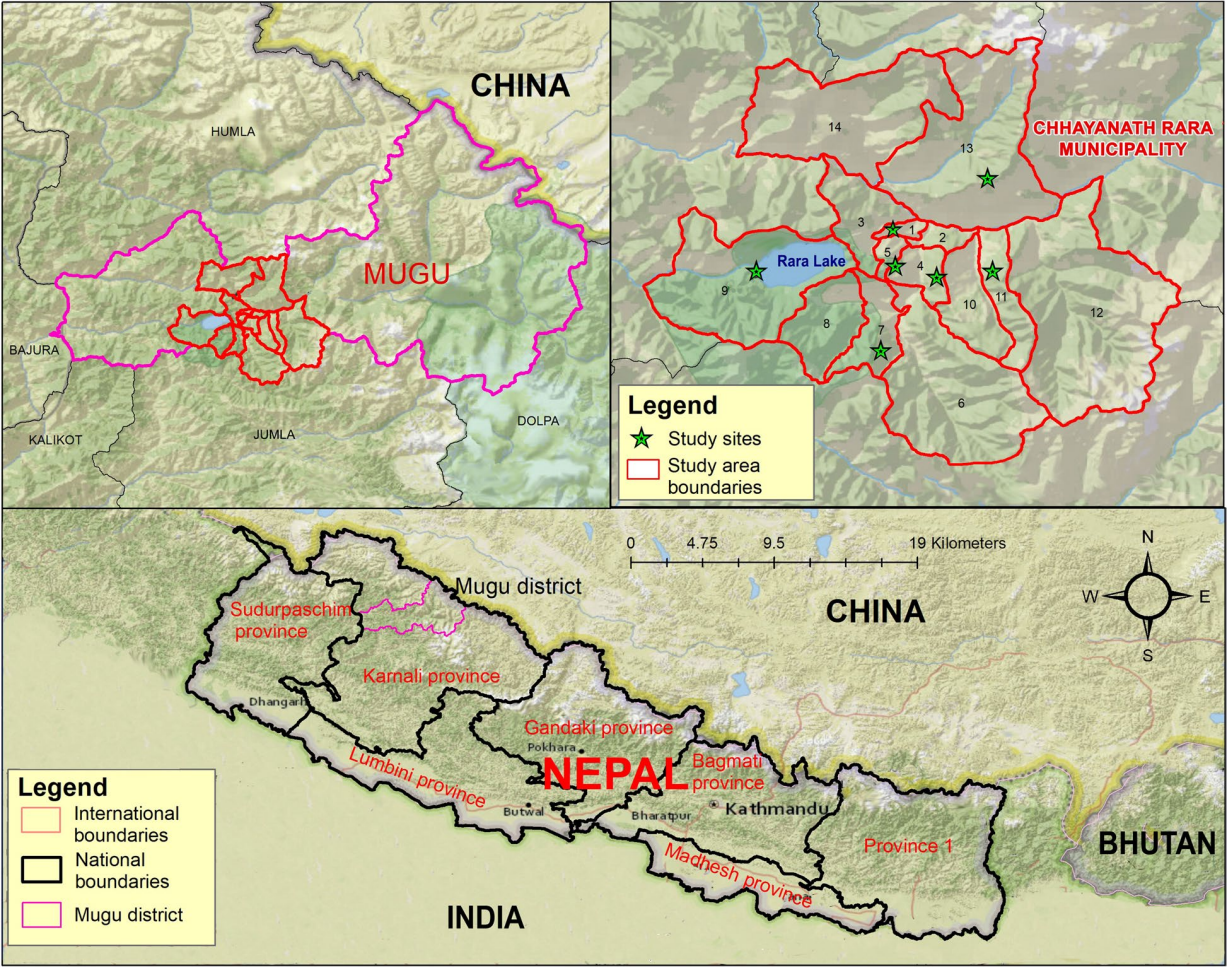
Map of study areas. The map was created using ArcGIS desktop version 10.8. The shape file of the administrative districts and location for Nepal was obtained from the Government of Nepal, Ministry of Land Management, and Survey Department website and were publicly available for unrestricted use (http://www.dos.gov.np/nepal-map)

A systematic random sampling technique was used to select the desired sample from a list of households with mothers who had at least one child aged 6 − 23 months. Seven of the fourteen wards (50%) were randomly selected for the study sites within the municipality **(**Fig. 1**)**. The list of households within the selected wards was obtained from the local Female Community Health Volunteers (FCHVs). Households were selected with a sampling interval of two, which was calculated from the list of 659 eligible households. From each household, only one eligible participant was chosen. If more than one mother were eligible to participate in the selected household, a lottery method was utilised to choose one participant from the household. In the absence of an eligible participant in the selected household, an eligible participant from an adjacent household was selected.

### Data collection

Data were collected using face-to-face interviews in the Nepali language. A semi-structured questionnaire was used for the interviews. The questionnaire consisted of five section: 1) socio demographic characteristic; 2) behavior factors; 3) child feeding practices; 4) dietary diversity; 5) nutrition allowance scheme information. Socio demographic and behavior related information was adapted from the Nepal Demographic and Health Survey, 2016 [28], Child feeding practices and dietary diversity tools were adapted from the Indicators for assessing infant and young child feeding practices: definitions and measurement methods. Geneva: World Health Organization (WHO) and the United Nations Children’s Fund (UNICEF) [29], and nutrition allowance scheme related questionnaire were prepared based on the literature review from the previously published reports [12, 19, 20]. All the tools were originally developed in the English language. Further, the tools were translated into the Nepali language and back-translated into English to ensure the validity and reliability of the tools. Nepali versions were pretested among 10% of the study sample (*n* = 32) in a neighbouring ward before the tools were used for data collection.

Interviews were conducted at the participants’ homes and lasted for up to 45 min. Trained enumerators who were public health undergraduates in their final year conducted the interviews. A field supervisor confirmed the quality of the data by cross-verifying the completed questionnaire on-site, and any discrepancies were discussed with enumerators.

### Outcome variables

Child feeding practices were the outcomes of interest in this study. In accordance with the updated indicators to assess infant and young child feeding (IYCF) practices at the household level recommended by the WHO and UNICEF, 2021, child feeding practices were defined as a child's Minimum Dietary Diversity (MDD), Minimum Meal Frequency (MMF), and Minimum Acceptable Diet (MAD) [29]. A 24-h dietary recall method was used to collect information on eight food groups following the WHO and unicef infant feeding guidelines. Consumed foods were grouped into eight categories: i) breast milk, ii) grains, roots, and tubers; iii) legumes and nuts; iiv) dairy products; v) flesh foods; vi) eggs; vii) vitamin A-rich fruits and vegetables, and viii) other fruits and vegetables to calculate dietary diversity [29].

Minimum dietary diversity was then calculated as the consumption of five or more food groups of the eight food groups during the recall day [29]. Minimum meal frequency was defined as breastfed and non-breastfed children who received solid, semi-solid, or soft foods for the minimum recommended number of times in the last 24-h. The minimum recommended feeding times are twice for breastfed infants 6 − 8 months; thrice for breastfed children 9–23 months, and four times for non-breastfeed children 6 − 23 months [29].

MAD was defined as i) the percentage of breastfed children 6–12 months of age who met both the minimum dietary diversity and the minimum meal frequency during the last 24- hours, and ii) non-breastfed children 6–23 months of age who received at least two milk feedings and had at least the minimum dietary diversity not including milk feeds and the minimum meal frequency during the previous 24 h [29].

Timely introduction of solid, semi-solid or soft foods: the proportion of infants 6 − 23 months of age who begins complementary food at six months [29]. Child feeding practice was considered appropriate if all the three indicators of the timely introduction of solid, complementary feeding, minimum dietary diversity, and minimum meal frequency were fulfilled [30, 31].

### Independent variables

#### Socio-demographic characteristics

Socio-demographic information such as mother’s age, ethnicity, religion, family type, mother’s and husband’s education, employment status, and family’s monthly income were collected. The indigenous castes such as *Janajati*, and *Dalit and minority* groups were included in disadvantaged ethnicity. While *Brahmin, and Chhetri/Thakuri* were categorised as non-disadvantaged groups [32]. Similarly, maternal factors such as antenatal check-ups (ANC), post-natal check-ups (PNC), age at marriage, age at first pregnancy, place of delivery, the distance to the nearest health facility, and place of child treatment were also collected.

### Nutritional allowance scheme

We also included information about timely payment of the nutrition allowance scheme; the amount of payment received, and expenditure behaviour, that influences child feeding practices [23].

### Data management and analysis

The collected data were entered in Epi Data version 3.1 and analysed using Stata/MP version 14.1 (Stata Corp LP, College Station, Texas). Descriptive statistics were used for summarising the outcome and independent variables. Continuous variables are presented as means and standard deviations (SD), after checking for normal distribution. The categorical variables are presented as percentages. For each outcome variable and independent variable, bivariate logistic regression models were performed to assess the association between independent and outcome variables. All independent variables from the bivariate analyses were included in the multivariable logistic regression models to control the potential confounders.

To prevent statistical bias in the multivariable logistic regression model, we examined multicollinearity among the independent variables using variation inflation factors (VIF). We used "10" as a cut-off value for the maximum level of VIF [33]. Results are presented as crude odds ratio (cOR) and adjusted odds ratio (aOR) with 95% confidence intervals (CIs). The *p*-values < 0.05 were considered statistically significant.

### Ethical consideration

The ethical clearance for this study was obtained from the Ethical Review Board (ERB) at the Nepal Health Research Council (Ref: 2788). Formal written approval was taken from the *Chhayanath Rara* Municipality office and health posts of selected wards to conduct this study. Informed written consent was obtained from the participants before proceeding to interviews. Participants were informed about voluntary participation, their right to refusal at any point, the confidentiality of the information shared, and the anonymity of their identity.

## Results

### Socio-demographic characteristics

Table 1 shows the socio-demographic information of the participants. More than half (59.1%) of the mothers were < 25 years of age and 44.9% of them did not have any formal school education. More than half (56.6%) of the mothers were < 20 years of age at marriage and their first pregnancy (50.9%). The majority of the mothers (90.6%) gave birth at a health facility centre and had completed recommended four ANC visits (90%).

**Table 1:** Demographic characteristics of the study participants (*n* = 318)

Socio-demographic characteristics	Frequency (n)	Percent (%)
**Ethnicity**		
Advantaged ethnic group	210	66.1
Disadvantaged ethnic group	108	33.9
**Father's employment**		
Paid	68	21.4
Unpaid	250	78.6
**Religion**		
Hindu	312	98.1
Non- Hindu	6	1.9
**Family type**		
Nuclear	106	33.3
Extended	212	66.7
**Family’s monthly income**		
Rs < 15 k (~ 150 USD)	228	71.7
Rs 15-50 k (~ 150- 500 USD)	90	28.3
**Distance to the nearest health facility (walking distance)**		
< 1 h	192	60.4
> 1 h	126	39.6
**Place for treatment of child's illnesses**		
Hospital	125	39.3
Health post	135	42.5
Traditional healers *(Dhami, Jhakri*)	58	18.2
**Sufficiency of food**		
6 months	231	72.6
12 months	63	19.8
More than 12 months	24	7.5
**Maternal factors**		
**Mothers’ age**		
Under 25 years	188	59.1
25 to 30 years	83	26.1
30 years and older	47	14.8
**Mothers’ education**		
No education	143	44.9
Primary	82	25.7
Secondary/higher	93	29.4
**Mother's employment**		
Paid	54	16.9
Unpaid	264	83.1
**Source of Income**		
Self-employed	30	9.5
Government job	44	13.8
Agriculture	244	76.7
**Age at marriage**		
< 20 years	180	56.6
> 20 years	138	43.4
**Age at first pregnancy**		
< 20 years	138	43.4
> 20 years	180	56.6
**Place of delivery**		
Home Delivery	30	9.4
Health facility	288	90.6
**ANC visit during last pregnancy**		
> 4 ANC visit	289	90.9
< 4 ANC visit	29	9.1
**Behavior related factors**		
**Current tobacco smoking behavior**		
Yes	38	11.9
No	280	88.1

For treatment of childhood illness, 42.5% of the mothers had taken their child to the health post and 18.2% of the mothers had taken their child to *Dhami* and *Jhakri* (traditional healers) **(**Table 1**)**.

Two-thirds (66.1%) of mothers were from the disadvantaged ethnic group and most (98.1%) followed Hinduism. The majority of the child’s mother (83.1%) and father (78.6%) were in unpaid employment. Three-fourths (71.7%) of the respondent’s monthly family income was NRs < 15,000 (~ < 150 USD). Two-thirds (66.7%) of the participants lived in an extended family. More than one-fourth (76.7%) of respondents had agriculture as their income source.

### Child characteristics and feeding practices

The mean (SD) age of children was 11.8 (5.3) months. Just over half (51.9%) of the children were female, and 59.4% were in the age group of 6 − 11 months. The majority of children (82.1%) were reported by mothers to have had normal birth weight.

The mean (SD) child’s dietary diversity score was 3.4 (1.1). Almost all children were consuming food from grains and legumes but < 30% were consuming dairy and vitamin A rich fruits and vegetables. Overall, 47.2% (95% CI: 41.7%, 52.7%) of the children aged 6–23 months were not consuming a diverse diet, 46.9% (95% CI: 41.4%, 52.4%) were not consuming recommended times (meal frequency) and 51.6% (46.1%, 57.1%) of the total children did not meet the minimum acceptable diet. Only 27.4% (95% CI: 22.7%, 32.5%) of the total children had appropriate complementary feeding practices **(**Table 2**)**.

**Table 2:** Child characteristics and nutrition status (*n* = 318)

Variable	Mean	SD
**Age of child in months**	11.8	5.3
**Minimum dietary diversity score**	3.4	1.1
**Child factors**	**Frequency (n)**	**Percent (%)**
**Sex of the child**		
Male	153	48.1
Female	165	51.9
**Age of child**		
6–11 months	189	59.4
12–17 months	65	20.4
18–23 months	64	20.2
**Childbirth weight**		
< 2.5 kg	57	17.9
> 2.5 kg	261	82.1
**Minimum dietary diversity score**		
Not diverse (< 4)	150	47.2
Diverse (≥ 4)	168	52.8
**Met recommended minimum meal frequency in last 24 h**		
No	149	46.9
Yes	169	53.1
**Minimum acceptable diet**		
Not met	164	51.7
Met	154	48.3
**Complementary feeding practices**		
Appropriate	87	27.4
Inappropriate	231	72.6

### Nutrition allowance scheme

Table 3 shows the information on the nutrition allowance. More than three-fourths (78%) of participants had received an allowance from the municipality, whereas 22% had received nutrition allowance schemes from the ward office. About (16.0%) of participants experienced inconvenience in receiving the nutrition allowance. The participants expressed that distance to the health facility (8.2%) and short time duration for collection of allowance (7.9%) were key reasons for facing inconvenience in receiving the allowance. Likewise, about two-thirds (66.4%) of participants mentioned that they were not getting the monthly nutrition allowance timely.

**Table 3:** Utilization of Nutrition allowance scheme (*n* = 318)

Variables	Frequency (n)	Percent (%)
**Receives nutrition allowance from**		
Local ward office	70	22.0
Municipality office	248	78.0
**Convenience in receiving allowance**		
Convenient	267	84.0
Inconvenient	51	16.0
**Challenges to receive allowance (*n* = 51)**		
Distance to facility	26	8.2
Limitation of time	25	7.9
**Support from stakeholders**		
Provide allowance timely	28	8.8
Provide information about the incentive	23	7.2
**Support from family members**		
Yes	315	99.1
No	3	.9
**Support got from family members to receive the allowance**		
Help to receive incentive	134	42.1
Help to buy nutritious food from allowance	184	57.9
**Hours engaged in household work**		
3 h	18	5.7
4 h	22	6.9
5 h	37	11.6
6 h and more	241	75.8
**Expenditure of allowance**		
Purchase food for child’s nutrition (nutritious food)	264	40.1
Household purchases	151	22.9
Purchase clothes for child	236	35.8
Recreational activities (parents expense their recreation activities)	8	1.2
**Expenditure of allowance on child nutrition**		
No	264	83.1
Yes	54	16.9

In terms of allowance expenditures, 40.1% of allowance was spent on child's nutrition such as purchasing nutritious food from the market, 35.8% on children's clothes, 22.9% on household purchases, and the remaining 1.2% spent on parents’ recreational activities **(**Table 3).

### Factors associated with child feeding practices

In the bivariate logistic regression analysis, several demographic and maternal-related factors were significantly associated with higher odds of inappropriate child feeding practices. Fathers in unpaid employment (cOR = 2.08; 95% CI: 1.18, 3.67) compared with paid employment, families with monthly income < 150 USD (cOR = 1.73; 95% CI: 1.02, 2.94) compared with an income of ≥ 150 USD, mothers in unpaid employment (cOR = 2.80; 95% CI: 1.53, 5.14) compared with paid employment, were found to have higher odds of inappropriate child feeding practices (Table 4).

**Table 4:** Bivariate and multivariable logistic regression analysis of factors affecting child feeding practices among children aged 6 − 23 months in remote Mugu district of Nepal (*n* = 318)

Variables	Child feeding practices		Bivariate model cOR (95% CI)	*P*-value^1^	Multivariable model aOR (95% CI)	*P*-value^2^
	**Appropriate n (%)**	**Inappropriate n (%)**				
**Socio-demographic characteristics**						
**Ethnicity**						
Advantaged ethnic group	62 (71.3)	148 (64.1)	Ref		Ref	
Disadvantaged ethnic group	25 (28.7)	83 (35.9)	1.39 (0.81–2.37)	0.228	1.22 (0.65–2.29)	0.528
**Father's occupation**						
Paid	27 (31.1)	41 (17.7)	Ref		Ref	
Unpaid	60 (68.9)	190 (82.3)	2.08 (1.18–3.67)	0.011*	1.18 (0.45–3.10)	0.723
**Family type**						
Nuclear	34 (39.1)	72 (31.2)	Ref		Ref	
Extended	53 (60.9)	159 (68.8)	1.41 (0.84–2.36)	0.183	1.45 (0.81–2.59)	0.203
**Family monthly income**						
Rs < 15 k (150 USD)	55 (63.2)	173 (74.9)	1.73 (1.02–2.94)	0.041*	1.19 (1.05–2.42)	0.045*
Rs 15-50 k (150- 500 USD)	32 (36.8)	58 (25.1)	Ref		Ref	
**Place of delivery**						
Home Delivery	4 (4.6)	26 (11.3)	2.63 (0.89–7.77)	0.080	4.70 (1.03–21.31)	0.045*
Health facility	83 (95.4)	205 (88.7)	Ref		Ref	
**Distance between house and nearest health facility**						
< 1 h	57 (65.5)	135 (58.4)	Ref		Ref	
> 1 h	30 (34.5)	96 (41.5)	1.35 (0.80–2.25)	0.251	1.60 (0.88–2.92)	0.119
**Place for child illness treatment**						
Hospital	39 (37.9)	92 (39.8)	Ref		Ref	
Health post	39 (44.8)	96 (41.6)	0.88 (0.51–1.52)	0.654	0.75 (0.40–1.37)	0.353
Traditional healers (*Dhami, jhakri*)	15 (17.3)	43 (18.6)	1.02 (0.50–2.09)	0.939	0.79 (0.36–1.72)	0.555
**Maternal factors**						
**Respondent’s age**						
Below 25	52 (59.8)	136 (58.9)	1.06 (0.60–1.88)	0.832	1.06 (0.52–2.14)	0.860
25 to 30	24 (27.6)	59 (25.5)	Ref		Ref	
30 and above	11 (12.6)	36 (15.6)	1.33 (0.58–3.03)	0.497	1.43 (0.60–3.40)	0.409
**Mothers’ education**						
No education	32 (36.78)	111 (48.1)	1.49 (0.82–2.70)	0.284	1.31 (0.39–4.38)	0.652
Primary	27 (31.1)	55 (23.8)	0.87 (0.46–1.66)	0.689	0.90 (0.27–3.00)	0.871
Secondary/higher	28 (32.1)	65 (28.1)	Ref		Ref	
**Mother's occupation**						
Paid	25 (28.7)	29 (12.5)	Ref		Ref	
Unpaid	62 (71.3)	202 (87.5)	2.80 (1.53–5.14)	0.001*	2.56 (1.06–6.19)	0.036*
**Mother age at marriage in years**						
< 20	45 (51.7)	135 (58.4)	Ref		Ref	
> 20	42 (48.3)	96 (41.5)	0.76 (0.46–1.25)	0.282	0.84 (0.34–2.05)	0.712
**Age at first pregnancy**						
< 20	33 (37.9)	105 (45.4)	1.36 (0.82–2.25)	0.228	1.09 (0.45–2.63)	0.841
> 20	54 (62.1)	126 (54.6)	Ref			
**ANC visit during last pregnancy**						
> 4 ANC visit	8 (9.2)	21 (9.1)	Ref		Ref	
< 4 ANC visit	79 (90.8)	210 (90.9)	0.98 (0.42–2.32)	0.977	0.31 (0.0.08–1.10)	0.070
**Child factors**						
**Sex of the child**						
Male	48 (55.2)	105 (45.5)	Ref		Ref	
Female	39 (44.8)	126 (54.5)	1.47 (0.89–2.42)	0.123	1.61 (0.93–2.77)	0.085
**Age of child**						
6–11 months	54 (62.1)	135 (58.4)	0.76 (0.39–1.47)	0.426	0.72 (0.35–1.48)	0.379
12–17 months	18 (20.7)	47 (20.4)	0.79 (0.36–1.76)	0.580	0.77 (0.31–1.91)	0.585
18–23 months	15 (17.2)	49 (21.2)	Ref		Ref	
**Childbirth weight**						
< 2.5 kg	14 (16.1)	43 (18.6)	1.19 (0.61–2.30)	0.601	1.18 (0.56–2.51)	0.651
> 2.5 kg	73 (83.9)	188 (81.4)	Ref		Ref	
**Expenditure of allowance on child nutrition**						
No	16 (18.4)	38 (16.4)	0.87 (0.45–1.66)	0.681	0.88 (0.44–1.77)	0.731
Yes	71 (81.6)	193 (83.6)	Ref		Ref	

*cOR* Crude odds ratio for unadjusted model, *aOR* adjusted odds ratio

^*^denotes for statistically significant at *p* < 0.05

^1^Bivariate binary logistic regression analysis

^2^Multivariable binary logistic regression analysis

Multivariable binary logistic regression shows that mothers who had home delivered their child (aOR = 4.70; 95% CI: 1.03, 21.31) compared to those who delivered their child in the health facility had higher odds of inappropriate child feeding practices. Children of mothers in unpaid work (aOR = 2.56; 95% CI: 1.06, 6.19) were 2.5 times more likely to have inappropriate child feeding practices compared to children of mothers in paid work. Children from households with a monthly income of < 150 USD (aOR = 1.19; 95% CI: 1.05, 2.42) compared to those ≥ 150 USD had increased odds of inappropriate child feeding practices. In the multivariable model, father employment status did not show a significant association with inappropriate feeding practices. We did not find an association between the expenditure of allowance on a child’s nutrition and feeding practices (Table 4).

## Discussion

The current study was designed to investigate child feeding practices among families receiving nutrition allowances scheme in rural Mugu, Nepal. This study provides evidence on the relationship of maternal factors, child-related factors, and utilisation of government-run nutrition allowance programme with child feeding practices.

This study depicts that child feeding practices are still low which is consistent with findings from other studies conducted in South Asia [34], and Africa [35, 36]. Our study shows that more than half of the children (52.8%) met meal diversity, over half meeting meal frequency and almost half (48.3%) had a minimum acceptable diet, higher than the national average Nepal Demographic and Health Survey (NDHS) report of 47% of children meeting the minimum dietary diversity and 36% meeting the minimum acceptable diet in 2016 [28]. This difference may be because of the nutrition allowance scheme or because NDHS 2016 was collected five years before this study. We are unable to test this assumption because of a lack of data. Other studies from Nepal reported a lower percentage of children meeting minimum diversity (33–35%), and minimum acceptable diet (29–33%) but a higher percentage of children meeting minimum meal frequency (83–84%) compared to our study findings [37, 38].

Our findings are also consistent with other studies from Madagascar [39] and South Asia [40] but contrary to studies from Ethiopia [41] and India [42] which reported only 23% met minimum dietary diversity. The mean (SD) child dietary diversity score in our study was 3.4 (1.1) which is greater than reported in the Indian National Family Health Survey and in a study from Ethiopia where the mean score was 2.26 [42], and 2.89 [41], respectively.

A financial benefit to the family in the form of a cash transfer has been recognized as a powerful strategy to improve child health and nutrition status, although with varied outcomes [22, 23]. This study found no association between child grant allowance expenditure on child nutrition with child feeding practices. The possible explanation for this might be a lack of knowledge of childhood nutrition and feeding practices among mothers who are the primary caretaker of children [16, 17, 43]. Community mobilization in creating awareness of the cash transfer programme and childhood nutrition by FCHVs could have helped in improving feeding practices [19].

The absence of an association between allowance expenditure on child nutrition and feeding practices indicates that access to resources may not improve child feeding practices if other determinants of child feeding practices such as nutrition knowledge, disease, infection, health care access, etc. are not addressed [22]. Therefore, offering a cash transfer alone is not adequate to improve child feeding practices without addressing other determinants including nutrition education and counselling [44].

A review of the child cash transfer programme reports that the cash transfer contributes to enhancing the economic status of the family and improved food consumption, resulting in improving the health and nutrition status of the children [24]. A study from Nepal also reported that the unconditional cash transfer programme helped to reduce childhood stunting, underweight, and wasting by 9.4%, 16.5%, and 5.1%, respectively in the Karnali province of Nepal [23]. The improvements in other indicators of childhood nutrition as found in these studies have different pathways to the outcomes they have measured. None of these studies reported child feeding practices and we are unable to make comparisons with these studies highlighting the research gap in nutrition allowance and feeding practices in Nepal.

Another systematic review showed that the cash transfer programmes reduced childhood illness by 2.8% [45], and children born in health care centres had better feeding practices resulting in reduced malnutrition than children born at home in Pakistan [46]. This finding is consistent with a previous study conducted in Ethiopia, where mothers who gave birth in hospitals were 2.4 times more likely than mothers who gave birth in health centres to have adequate feeding practices [35]. This could be because women who had more facility-based deliveries were more likely to receive an education on proper feeding practices during their visit [47]. We found similar findings in our study, where children of mothers who had delivered at home were at increased odds of inappropriate child feeding practices compared to mothers who delivered at a health facility. This finding however should be interpreted with caution because of the wider confidence interval.

We did not observe the association between ANC visits and feeding practices. Despite Nepal’s government policy for nutrition counselling during ANC, the lack of nutrition counselling among mothers at the health centres in the study could explain the absence of association between ANC visits and feeding practices. In our study, children from lower-income families had higher odds of inappropriate child feeding practices which is consistent with findings from other studies in South Asia [46], Southern Ethiopia [36], and Tanzania [48]. Children living in poverty are prone to deprivation such as lack of food, clothes, health facilities, etc. which makes them vulnerable to poor health and undernutrition [12].

Children of mothers in unpaid employment mainly in agriculture had higher odds of inappropriate feeding practices. This finding is consistent with prior research, which found that increased workload and poor income inhibit mothers to establish and maintain healthy child feeding habits [34]. The mothers in our study have the double burden of unpaid employment and being involved in agriculture. Even though we were unable to measure workload, Nepali mothers are restricted by household chores and have limited time for themselves or to spend with their child and prepare for appropriate child feeding practices [37]. Nepali mothers are the primary caretakers of infants, the elderly, and the sick, primary cook, and perform all household work. Previous studies have shown that mothers who are employed are more likely to provide a minimum acceptable diet to their children [49]. Likewise, the previous studies have also reported that employed mothers are a protective factor against inadequate dietary diversity and inadequate meal frequency in Sri Lanka and India, respectively [50]. Analysis of cash transfer programme around Asia, Latin America, and Africa shows that when the majority of cash transfer was spent on nutrition, household food consumption increased which can directly add value to appropriate feeding practice [24, 51]. The conditional usage of nutrition allowance in the programme could have helped in controlling expenditure on items not associated with child nutrition in our study sample.

Despite the fact that the nutrition allowance scheme was implemented in Mugu, Nepal, child feeding practices were suboptimal. This study found several factors associated with inappropriate feeding practices, which should be considered in designing programmes targeted at improving child feeding practices in remote Nepal. Programmes should be targeted at children of mothers who opt for home delivery, lower-income families, and children of mothers in unpaid employment. Child feeding practices do not improve when cash transfer programs are not integrated with other programs. The Government of Nepal should integrate the nutrition allowance programme with comprehensive nutrition-sensitive and specific interventions backed by the theory of behavior change framework. Also, the findings indicate that several national and international agencies working on improving childhood nutrition in Nepal should cooperate in delivering a cost-effective nutrition intervention. In addition to these, the provision of monitoring mechanism for utilization of nutrition allowance is inevitable for effective implementation of child cash transfer program in Nepal. It may be possible to mobilize female community health volunteers in the targeted district of Nepal to closely monitor and reinforce the key themes of the cash transfer program, which can enables the program's better execution. A training program for FCHVs on infant young child feeding (IYCF) counselling was conducted in Nepal in order to enhance social behavior change communication [52]. In Nepal, FCHVs have been effective in contributing significantly to a range of community-based mother and child programs in a variety of roles as health promoters, dispensers, and service providers [53]. Future research can build on the findings of this study to determine the impact of the cash transfer program on improving child feeding practices among intended beneficiaries in cash transfer program implemented districts of Nepal. Likewise, this study provides the basis for conducting the qualitative explorations for poor utilization of government implemented nutrition allowance scheme on child nutrition.

Our study has some limitations. First, due to the cross-sectional study design of the study, the directional cause and effect relationship between independent and dependent variables cannot be inferred. Second, confinement of the sample size for this study to a single season may limit the generalizability of findings to other seasons as child dietary diversity is influenced by seasonal variability in food. Third, recall and social desirability bias may have occurred during the interview. Fourth, a single 24-h recall was used, which does not accurately reflect the respondents' usual dietary habits.

## Conclusions

The current study found that children's feeding practices are still unsatisfactory in the remote Mugu district in the Himalayan region of Nepal. Home delivery, lower-income households, and mothers who were unpaid workers were found to be significantly associated with poor child feeding practices. This study found no significant association between spending allowance on a child's feeding practices. The concerned stakeholders and health workers should strengthen the system and procedure for early counselling to impart knowledge on the importance of appropriate child feeding practices and its outcomes. Along with the child cash transfer programme, context-specific behaviour change strategies are needed to improve child feeding practices.

## Supplementary Information

**Additional file 1.** Dataset.

**Additional file 2.** Questionnaire (English versions).

**Additional file 3.** Questionnaire (Nepali versions).

**Additional file 4.** STROBE Statement.

## Data Availability

All relevant data are within the manuscript and its supporting information files.

## Abbreviations

ANC: Antenatal checkup
aOR: Adjusted odds ratio
CI: Confidence interval
cOR: Crude odds ratio
DHSs: Demographic and health surveys
FCHVs: Female community health volunteers
ERB: Ethical review board
IYCF: Infant and young child feeding
LMICs: Low and middle-income countries
MAD: Minimum acceptable diet
MDD: Minimum dietary diversity
MMF: Minimum meal frequency
VIF: Variation inflation factor
WHO: World health organization
