# Supplementary material for: The factors influencing inappropriate child feeding practices among families receiving nutrition allowance in the Himalayan region of Nepal
Source: BMC Nutr. 2023 Feb 20;9:33. doi: 10.1186/s40795-023-00691-3 (PMC9940375; doi:10.1186/s40795-023-00691-3)
Supplement: Supplementary file 2 — Additional file 2. Questionnaire (English versions). [file 40795_2023_691_MOESM2_ESM.pdf]

The factors influencing inappropriate child feeding practices among families receiving nutrition allowance in the Himalayan region of Nepal

Section 1 A: Socio demographic Characteristics

S.N	Question	Response
1	Sex	a) Male<br>b) Female
2	How old are you?	
3	What is the highest level of education you have completed?	a) No formal schooling<br>b) Less than primary school<br>c) Primary school completed Secondary school completed<br>d) Higher secondary (10+2)<br>e) Above higher secondary
4	What is your ethnic background?	f) Brahmin<br>g) Kshetri / Thakuri<br>h) Janjati<br>i) Dalit<br>j) Others
5	What is your occupation?	k) Government employee<br>l) Non-government employee<br>m) Self-employed<br>n) Student<br>o) Homemaker<br>p) Unemployed (unable to work)
6	What is your Husband Occupation?	
7	How many members are there in your house?	
8	What is your religion?	a) Hindu<br>b) Buddhist<br>c) Ishlam<br>d) Christianity<br>e) Others
9	What is your income source?	f) Business<br>g) Teaching<br>h) Agriculture<br>i) Others …….

10	How much income in your family per month (in Rs.)	
11	What was your Age at marriage?	
12	What was your Age at first pregnancy?	
13	Where did you delivery your baby?	a) Health facilities<br>b) Home
14	How many times did you attend ANC when you were pregnant?	
15	How many times did you attend PNC after delivery of child?	
16	What was your last child's birth weight?	a) less than 1.5 kg VLBW<br>b) 1.5- 2.5kg LBW<br>c) 2.5- 4 kg Normal<br>d) more than 4 kg Large birth weight
17	How many children have you ever born?	
18	What is the distance between your house and nearest health facility?	a) less than 30 minutes<br>b) 30 minutes to 1 hour<br>c) 1-2 hours<br>d) more than 2 hours
19	What is the place for your child illness treatment?	a) Hospital (District, Zonal, Tertiary care)<br>b) Health Post<br>c) Dhami, jhakri<br>d) Others
20	For how many months, Food is sufficient for your family members?	a. <6 months<br>b. <12 months<br>c. >12 months
21	Household ownership of agriculture	a) Yes<br>b) No

Section 2 B. Behaviors Related Information

S.N	Question	Response
2.1	Do you currently smoke any tobacco products, such as cigarettes, cigars, pipes, bidis, hukahs or tamakhus?	a) Yes<br>b) No, if no go to another question
2.2	How old were you when you first started smoking?	
2.3	Do you smoke any tobacco products currently?	

2.4	Have you ever consumed any alcoholic drink such as beer, wine, spirits, fermented cider or (jaad, raksi, tungba?	a) Yes<br>b) If No, go to another question
2.5	How frequently have you had at least one alcoholic drink?	
2.6	Have you consumed alcoholic drink within the past 7 days?	a) Yes<br>b) No if No go to another question
2.7	During the past 7 days, when you consumed alcoholic drink, how often was it with meals?	a) Usually with meals<br>b) Sometimes with meals<br>c) Rarely with meals<br>d) Never with meals
2.8	What is your food habit?	a)Vegetarian<br>b) Non vegetarian

Section 3 C: Child Feeding Practices

3a. Timely introduction of complementary foods		
3.1	Decision makers on child feeding	a) Husband<br>b) Wife<br>c) Both<br>d) Others
3.2	Have you introduced any food apart from breast milk to your child?	a) Yes<br>No (if no move to other question.
3.3	At what age did you introduce any others foods apart from breast milk to your child?	…………….. Months
3.4	Is your still child breast feeding?	a) Yes<br>b) No
3.5	If no, why?	
3.6	What was your reason for introducing food to your child?	a) Breast milk was no longer sufficient<br>b) Because child was 6 month old<br>c) other specify
3.7	How long after birth did you first put to the breast?	A) Immediately<br>B) ….. Hours<br>C) …….Days

3b. Dietary Diversity

From the meals mentioned, indicate if child receive any of these liquids and foods yesterday

**Food group**		**Example**	**Yes**	**No**
3.8	Breast milk	Continuously breastfeed		
3.9	Eggs	Eggs		
3.10	Grains, roots	Noodles, Rice, Potatoes, Biscuits, Bread, Barley, Maize, Soya bean.		
3.11	Legumes and Nuts	Beans, Groundnuts, Okhar Grams, Bengal , Peas , Rajma		
3.12	Flesh foods	Chicken, Goat , Kidney liver , Fish ,Sheep		
3.13	Dairy products	Milk , Yoghurt Ghee, Panir,		
3.14	Vitamin A rich Fruits and Vegetables	Carrot , Apple, Potato, Cucumber , Radish DGLV		
3.15	Others Fruits and vegetables	Any others fruit and vegetables (Tomato, Pumpkin, Green bean, Cabbage, Carrot, Cucumber, potato, Sweet potato, Spinach Cauliflower.		

3c. Minimum Meal Frequency

3.16	How many times do you feed your child a day?	a) 2 times<br>b) 3 times<br>c) 4 times or more

4a. Nutrition allowance scheme information

4.1	Have you receive nutrition allowance schemes?	a) Yes<br>b) No
4.2	Where do you get a nutrition allowance schemes?	a) Ward office<br>b) Municipality<br>c) Bank<br>d) Others
4.3	How much incentive is received as Allowance schemes?	………………………

4.4	From where you have heard about nutrition allowance schemes?	a) Newspaper<br>b) T.V<br>c) Public Representative<br>d) Others
4.5	Who goes to receive the incentive?	a) Self<br>b) Family other members
4.6	How convenient it was to receive incentive earlier?	a) Convenient<br>b) Inconvenient
4.7	If Inconvenient, then what are the difficulties you have to face?	c)
4.8	Was there any role of stakeholders to distribute the incentive receive by you?	a) Yes<br>b) No
4.9	If yes then what are the support you get from stakeholder?	
4.10	If No, then what are the difficulties you have to face to receive allowance?	
4.11	Have you being support by your family members to receive nutrition allowance schemes?	a) Yes<br>b) No
4.12	If yes then what are the support you get from your family members?	
4.13	Have you engaged in professional /household works?	a) Yes<br>b) No
4.14	If yes then how many hours do you engaged in those works?	a) 3 hours<br>b) 4 hours<br>c) 5 hours<br>d) 6-8 hours and more
4.15	Being engaged in your own profession have you get time to receive incentive?	a) Yes<br>b) No
4.16	Where do you spend the incentive?	c) Children Nutrition<br>d) Household activities<br>e) Children Clothes<br>a) Others (Play group school, Recreation activities)
