# Supplementary material for: The factors influencing inappropriate child feeding practices among families receiving nutrition allowance in the Himalayan region of Nepal
Source: BMC Nutr. 2023 Feb 20;9:33. doi: 10.1186/s40795-023-00691-3 (PMC9940375; doi:10.1186/s40795-023-00691-3)
Supplement: Supplementary file 3 — Additional file 3. Questionnaire (Nepali versions). [file 40795_2023_691_MOESM3_ESM.pdf]

प्रश्नावली

फाराम नं. ....   मितिः ....../......./२०७७

अन्तर्वार्ता दिने व्यक्तिको नामः ....   जिल्लाः ....

(महानगर/उपमहानगर/नगर/गाउँ)पालिका ....वार्ड नं.: ....

१. सामाजिक तथा जनसांङ्ख्यिक विवरणः

क.स.	प्रश्नावलि	प्रतिकिया
१	लिङ्ग	क) पुरुष<br>ख) महिला<br>ग) तेस्रो लिङ्गी
२	तपाइको उमेर कति हो ?	
३	तपाइले उत्तिर्ण गर्नु भएको उच्चतम शिक्षा कति हो ?	क) निरक्षर<br>ख) साक्षर<br>ग) आधारभूत<br>घ) मा.वि. तह<br>ङ) उच्चशिक्षा<br>१) स्नातक<br>२) स्नातकोत्तर<br>३) बिशेषज्ञ
४	तपाइको जातिय परिबेश के हो ?	क) ब्राहमण<br>ख) ठकुरी/ क्षेत्री<br>ग) दलित<br>घ) जनजाती<br>ङ अन्य
५	तपाई कुन् पेशा अपनाउनु भएको छ ?	क) व्यापार

		ख) सेवा, नोकरी<br>ग) कृषि<br>घ) स्व-रोजगार<br>ङ) विद्यार्थी<br>च) उद्यमी<br>ज) बेरोजगार<br>झ) अन्य ....
६	तपाई को श्रीमान को पेसा के हो?	....
७	तपाईको परिवार मा कति जना सदस्य छन् ?	.... जना
८	तपाई कुन् धर्म मान्नु हुन्छ ?	क) हिन्दु<br>ख) बौद्ध<br>ग) इसाई<br>च) अन्य ....
९	तपाईको आर्थिक स्रोत के हो ?	क) व्यापार<br>ख) सेवा, नोकरी<br>ग) कृषि<br>घ) बैदेशिक रोजगारी<br>ङ) अन्य ....
१०	तपाईको परिवारको मासिक आम्दानी कति हो ?	....
११	तपाई बिवाह गर्दा कति वर्ष को हुनुहुन्थ्यो?	...........बर्ष
१२	तपाई को पहिलो सन्तान जन्माउदा कति वर्ष को हुनुहुन्थ्यो?	...........बर्ष
१३	तपाई ले सन्तान जन्माएको स्थान कहाँ हो?	क) घर<br>ख) स्वास्थ्य सँस्था
१४	तपाई को अन्तिम सन्तान गर्व मा हुदा कति चोटी स्वास्थ्य सँस्था चेक जाच गर्न को लागि जानुभयो?	............पटक
१५	तपाई को अन्तिम सन्तानको जन्म भई सके पश्च्यात कति चोटी स्वास्थ्य सँस्था चेकजाच गर्न को लागि जानुभयो?	............पटक

१६	तपाई को अन्तिम सन्तानको तौल कति थियो?	क) १.५ किलो भन्दा कम<br>ख) १.५ देखि २.५ किलो<br>ग) २.५ देखि ४ किलो सम्म<br>घ) ४ किलो भन्दा माथि
१७	तपाई का जम्मा कति वटा सन्तान छन्?	
१८	तपाई घर देखि स्वास्थ्य सँस्था सम्म जान कति समय लाग्छ?	क) ३० मिनेट भन्दा कम<br>ख) ३० मिनेट देखि १ घण्टा<br>ग) १ घण्टा देखि २ घण्टा<br>घ) २ घण्टा भन्दा बढि
१९	बच्चाको रोग को उपचार गर्न कहाँ जानु हुन्छ?	क) अस्पताल(जिल्ला, अञ्चल, तिर्तयक केन्द्र)<br>ख) स्वास्थ्यचौकि<br>ग) धामि, झाकि<br>घ) अन्य
२०	तपाई को आम्दानीको श्रोत ले कति महिना सम्मको लागि खान पुग्छ?	क) ६ महिना<br>ख) १२ महिना<br>ग) १२ महिना भन्दा बढि
२१	तपाई को प्रमुख पेसा कृषि हो?	क) हो<br>ख) होइन

२ जीवनशैली सम्बन्धी जानकारी

क.स.	प्रश्नावली	प्रतिकिया
२.१	हाल तपाई कुनै प्रकारको सूर्तिजन्य पदार्थ जस्तैः चुरोट, सिगार, बिँडी, हुक्का वा तमाखु सेवन गर्नुहुन्छ ?	क) गर्छु<br>ख) गर्दैन (यदिगर्दैन भने प्र.नं.४ माजानुहोस्)

२.२	पहिलो पटक सूर्तिजन्यपदार्थ सेवन गर्दा कति उमेर को हुनुहन्थ्यो ?	
२.३	हाल तपाईले कुनै किसिमका सूर्तिजन्य पदार्थ सेवन गर्नुहुन्छ ?	क) गर्छु<br>ख) गर्दैन
२.४	तपाईले मद्यपान गर्नुभएको छ ?	क) गर्छु<br>ख)गर्दैन(यदि गर्दैन भने तेस्रो तहको प्रश्न मा जानुहोस्)
२.५	तपाई कति समयको अन्तरालमा कम्तिमा एक प्रकारको मद्यपान गर्नुहुन्छ ?	
२.६	तपाईले कुनै विगत ७ दिन भित्रमा मद्यपान गर्नुभएको छ ?	क) गरेको छु<br>ख) गरेको छैन
२.७	तपाईले कुनै विगत ७ दिन भित्र मद्यपान गर्दा कतिपटक खानासंगै मद्यपान गर्नुभयो ?	क) प्राय<br>ख) कहिलेकाहीँ<br>ग) एकदमै कम<br>घ) कहिल्यै पनिलिदैन
२.८	तपाई को खानाको प्रकृति के हो?	क) साकाहारी<br>ख) माँसाहारी

३. बच्चाको आहार सम्बन्धि जानकारी

**१.पूरक खानाबारेमा**		
३.१	पुरक खाना खुवाउनमा कस्ले निणय गर्दछ?	क) बुवा<br>ख) आमा<br>ग) अरु
३.२	तपाई ले आमाको दुध बाहेक अन्य खानाखुवाउनु भएको छ?	क) छ<br>ख) छैन - यदि छैन भने प्र.न १.४)

३.३	यदि छ भने कति उमेर हुदा देखि खुवाउन सुरु गर्नु भयो?	..........महिना
३.४	तपाई को बच्चा ले अझै आमाको दुध खान्छ?	क) खान्छ<br><br>ख) खादैन
३.५	यदि खादैन भने किन खुवाउनु भएन?	....
३.६	तपाइले बच्चालाई पुरक खाना खुवाउनु को कारण के हो?	क) आमाको दुध ले मात्रनपुग्ने भएर<br><br>ख)बच्चा ६ महिना पुरा भएकोले<br><br>ग) अन्यकारण
३.७	बच्चा जन्मेको कति समय मा आमाको दुध खुवाउनु भयो?	क) तत्कालै<br><br>ख) ....घण्टा<br><br>ग)......दिन

२. आहार बिबिधता

**खानाको समुह**		**उधारण**	**खाए**	**खाएन**
३.८	अण्डा	अण्डा		
३.९	अन्न, जरा	चाउचाउ,जौ,मकै,सोयाबिन, भात, आलु, बिस्कुट,रोटी		
३.१०	फलिया, बदाम	सिमि,ओखर,बदाम, ग्राम, मटर, राजमा		
३.११	मासु खाना	माछा, कलेजो खसी, बोका।		
३.१२	दुधका प्कार	दहि, घिउ,दुध, पनिर।		
३.१३	फलफुल र तरकारि काप्रकार	स्याउ,गाजर, मुला, काक्रा		
३.१४	अरु फलफुल र सागपात	बोडी, बन्दा, मिठो आलु, पालङ्गो, काउली		

३. न्युनतम् खानाआबृर्ति

३.१५	दिनमा कति पटक बच्चालाई पुरक खानाखुवाउनु हुन्छ?	क) २ पटक<br>ख) ३ पटक<br>ग) ४ वा सो भन्दा बढि

४. पोषणभत्ता सम्बन्धित प्रश्नावलि

क.सं.	प्रश्नहरु	प्रतिक्रिया
४.१	तपाई ले पोषण भत्तापाउनु हुन्छ?	क) पाउछु<br>ख) पाउदैन
४.२	तपाई ले पोषण भत्ताकहाँ बाट प्राप्तगर्नु हुन्छ?	क)वडा कार्यालय बाट<br>ख) गा.पा / नगरपालिका बाट<br>ग)बैंक<br>घ) अन्य.................
४.३	तपाईले पोषण भत्ता वाफत पाउने रकम कति हो?	
४.४	तपाईले पोषण भत्ता कार्यकम बारे कहाँ बाट जानकारी पाउनु भयो?	क) पत्रपत्रिका<br>ख) टि.भि<br>ग) जनप्रतिनिधि<br>घ) गा.पा

४.५	उक्तपोषण भत्ता प्राप्त गर्न को जानुहुन्छ ?	क) आफै<br><br>ख) घर को अरु सदस्य
४.६	सुरुवाती दिनहरु मा उक्त सेवा लिन तपाइलाई कतिको सहजभयो?	क) सहजभयो<br><br>ख) सहजभयन
४.७	यदि सहज भएन भने कस्तो किसिमको कठिनाइ भोग्नु भयो?	……………
४.८	उक्त सेवादिन र लिनमा समाजका अरु ब्यक्तिहरु (जनप्रतिनिधि, सामाजिक अभियान्ता, सरकारि तथा गैरसरकारि संस्था) को भुमिका थियो कि थिएन?	क) थियो<br>ख) थिएन
४.९	यदि भुमिका थियो भने कस्तो किसिमको थियो?	………………………
४.१०	यदि भुमिका थिएन भने कस्तो किसिमको समस्याहरु भोग्नु भयो?	……………………………
४.११	पोषण भत्ता प्राप्त गर्नु मा तपाईलाई तपाई को परिवार वाट सहयोग पाई राख्नू को छ ?	क) छ<br><br>ख) छैन
४.१२	यदि सहयोग पाई राख्नू भएको छ भने कस्ता किसिमका सहयोग पाउनु भएको छ ?	………
४.१३	तपाई आफै कुनै पेसा (जागिर अथवा अरु घरायसी काम) सँग आवद्धित हुनुहुन्छ ?	क) छु<br><br>ख) छैन
४.१४	हुनुहुन्छ भने दिनमा कति घण्टा जति काम गर्नु हुन्छ?	…………घण्टा
४.१५	तपाई आफै पेसा सँग आबद्धित भएर पोषण भत्ता प्राप्त गर्नमा समय पुगेको छ?	क) छ<br><br>ख) छैन
४.१६	तपाईले प्राप्त गर्नु भएको पैसा कुन कुन प्रयोजनमा प्रयोग गर्नु हुन्छ?	क) बच्चाको पोषणमा<br><br>ख) घरायसी काममा<br><br>ग) बच्चाको लुगा कपडामा<br><br>घ) अन्य
